# Technical and clinical validation of a novel plasma p‐tau217 assay: evaluation in real world population‐based and racially‐diverse cohorts

**DOI:** 10.1002/alz.092598

**Published:** 2025-01-09

**Authors:** Anuradha Sehrawat, Xuemei Zeng, Yijun Chen, Tara K Lafferty, Pamela C.L. Ferreira, Bruna Bellaver, Guilherme Povala, Tharick A. Pascoal, M. Ilyas Kamboh, Alexandra Gogola, Mary Ganguli, Eric E Abrahamson, Oscar L. Lopez, Victor L Villemagne, Victor L Villemagne, Milos D Ikonomovic, Beth E. Snitz, Ann D Cohen, Thomas K Karikari

**Affiliations:** ^1^ University of Pittsburgh, Pittsburgh, PA USA; ^2^ Department of Psychiatry, University of Pittsburgh School of Medicine, Pittsburgh, PA USA; ^3^ Departments of Psychiatry and Neurology, University of Pittsburgh School of Medicine, Pittsburgh, PA USA; ^4^ University of Pittsburgh Alzheimer's Disease Research Center, Pittsburgh, PA USA; ^5^ Division of Geriatrics & Neuropsychiatry, Department of Psychiatry, University of Pittsburgh, Pittsburgh, PA USA; ^6^ University of Pittsburgh School of Medicine, Pittsburgh, PA USA; ^7^ School of Medicine, University of Pittsburgh, Pittsburgh, PA USA; ^8^ University of Pittsburgh Alzheimer's Disease Research Center (ADRC), Pittsburgh, PA USA; ^9^ Department of Psychiatry, School of Medicine, University of Pittsburgh, Pittsburgh, PA USA

## Abstract

**Background:**

Plasma phospho‐tau217 (p‐tau217) is a promising blood‐based biomarkers for Alzheimer's disease (AD). However, the accessibility of pTau217 tests for both research and clinical applications has been constrained. Previous studies focused on highly‐phenotyped cohorts that differ substantially from the wider population. To broaden the access to this highly precise biomarker for AD, we developed a novel blood‐based immunoassay for p‐tau217 at the University of Pittsburgh (Pitt‐p‐tau217). Following thorough analytical validation, we evaluated the clinical utility of the assay in four independent real‐world cohorts (n=503 total) reflective of the wider population.

**Methods:**

A two‐step ultra‐sensitive p‐tau217 immunoassay was developed on the Quanterix HDX Simoa platform. Analytical validation focused on dilution linearity, competitive binding, day‐to‐day stability, and spike recovery. Clinical validation was performed in four cohorts including: two population‐based, racially‐diverse cohorts in medically underserved communities (the MYHAT‐Neuroimaging and Human Connectome cohorts); and a cohort of neuropathologically‐confirmed familial AD participants carrying pathogenic PSEN1 mutations. The fourth cohort included older adults >65 years attending a memory clinic. Assay performance was compared with the commercially‐available ALZpath‐p‐tau217 assay.

**Results:**

The new plasma Pitt‐p‐tau217 assay demonstrated high between‐run stability, linearity of dilution, and substantial signal reduction when a p‐tau217‐positive antigen was allowed to competitively bind to the p‐tau217 target in plasma samples before assay measurement. Clinically, the assay differentiated amyloid‐beta (Aβ) PET‐positive and ‐negative cognitively unimpaired individuals with areas under the curve (AUCs) of 0.84‐0.87, equivalent to 0.87‐0.90 for ALZPath and correlations of 0.40‐0.73. According to centiloids (CL), the Pitt‐p‐tau217 assay showed stepwise higher values from Aβ‐negative (CL<15) to low‐burden Aβ (CL15‐25) and Aβ‐positive (CL>25). By tau‐PET status, Pitt‐p‐tau217 was elevated in tau‐PET‐positive versus negative (P<0.001) and separated the groups with the same AUC of 0.71 as ALZpath‐p‐tau217. Furthermore, Pitt‐p‐tau217 was 5.3‐fold and 3.3‐fold higher in neuropathologically‐confirmed PSEN1 mutation carriers and sporadic AD participants respectively versus controls, with correlation of 0.93 with ALZpath‐p‐tau217.

**Conclusion:**

Our results indicate that the newly‐developed Pitt‐p‐tau217 assay, the first from an academic laboratory in North America, exhibits reliable and reproducible diagnostic accuracy for identifying individuals with biological evidence of AD. Demonstration of assay performance in inclusive cohorts suggests its suitability for use in the general population.

